# The presence of postmenopausal bleeding as prognostic parameter in patients with endometrial cancer: a retrospective multi-center study

**DOI:** 10.1186/1471-2407-9-460

**Published:** 2009-12-22

**Authors:** Veronika Seebacher, Maximilian Schmid, Stephan Polterauer, Katrin Hefler-Frischmuth, Heinz Leipold, Nicole Concin, Alexander Reinthaller, Lukas Hefler

**Affiliations:** 1 Department of Obstetrics & Gynecology, Medical University of Vienna, Währinger Gürtel 18-20, 1090 Vienna, Austria; 2 Department of Laboratory Medicine, Wilhelminenspital, Montleartstraße 37, 1160 Vienna, Austria; 3 Department of Obstetrics & Gynecology, Landeskrankenhaus Klagenfurt, St. Veiter Strasse 47, 9020 Klagenfurt, Austria; 4 Department of Obstetrics & Gynecology, Innsbruck Medical University, Christoph-Probst-Platz, Innrain 52, 6020 Innsbruck, Austria

## Abstract

**Background:**

To date, there is no consensus on the utility of screening procedures for the early detection of endometrial cancer. The value of transvaginal ultrasound for screening of asymptomatic endometrial cancer has been discussed controversially. This study was conducted to evaluate whether asymptomatic patients with endometrial cancer have a better prognosis than symptomatic patients with endometrial cancer diagnosed after postmenopausal bleeding.

**Methods:**

In the present multi-center study, the effect of the presence of postmenopausal bleeding on prognosis was evaluated retrospectively in 605 patients with endometrial cancer using patients' files. 543 patients (133 patients were asymptomatic, 410 patients were symptomatic) with endometrioid endometrial cancer were enrolled in all further analysis. Student's t-test, Cox regression analysis and Kaplan-Meier analysis were used were appropriate.

**Results:**

Presence/absence of a postmenopausal bleeding was not associated with tumor stage (p = 0.2) and age at diagnosis (p = 0.5). Asymptomatic patients with endometrial cancer had a significantly higher rate of well and moderate-differentiated tumors compared to symptomatic patients (p = 0.008). In univariable and multivariable survival analysis, tumor stage, tumor grade, and patients' age at diagnosis, but not presence/absence of a postmenopausal bleeding, were associated with disease free and overall survival.

**Conclusion:**

Asymptomatic patients with endometrial cancer have a higher rate of well differentiated tumors compared to patients with a postmenopausal bleeding prior to diagnosis. The prognosis of both groups of patients was similar.

## Background

Endometrial cancer is the most common gynecological malignancy in developed countries with an estimated 39,000 newly diagnosed cases in the United States in 2007. Although many patients are diagnosed with an early stage disease, 7400 deaths will occur [1].

Approximately 90% of women with endometrial cancer have abnormal uterine bleeding as the only presenting complaint leading to the diagnosis of the disease [2]. To date, there is no consensus on the utility of screening procedures for the early detection of endometrial cancer [3-8]. It has been suggested that screening of asymptomatic endometrial cancer by transvaginal ultrasound before the onset of clinical symptoms, i.e., postmenopausal bleeding, leads to an earlier diagnosis [8]. As in other malignancies, it can be hypothesized that an earlier diagnosis leads to a lower stage of disease, a less radical surgery, a therapy with less side effects and, subsequently, a better prognosis of affected patients.

Interestingly, relatively few studies are available investigating whether asymptomatic patients with endometrial cancer have a better prognosis than those detected after the onset of a postmenopausal bleeding. Gerber *et al*. performed a retrospective study in 190 postmenopausal patients with symptomatic endometrial cancer and 16 asymptomatic patients showing no difference in survival in this relatively small series [7]. Osmer *et al. *compared 61 and 22 postmenopausal women with symptomatic and asymptomatic endometrial cancer, respectively. Patients with asymptomatic endometrial cancers showed a less myometrial tumor infiltration and more well differentiated tumors than symptomatic patients. A better prognosis for asymptomatic patients has been suggested [8]. Kimura *et al*. reviewed a total of 304 endometrial cancer patients [9]. Patients diagnosed with asymptomatic endometrial cancer showed a significantly better 5-year overall survival rate than the patients diagnosed after the onset of postmenopausal bleeding. The distribution of clinical stages and histological grades did not differ between both groups.

The aim of the present study was to estimate whether asymptomatic patients with endometrial cancer have a better prognosis than symptomatic patients with endometrial cancer diagnosed after postmenopausal bleeding in a large series of postmenopausal patients with endometrial cancer in a multi-center study.

## Methods

### Patients

Clinical data were obtained retrospectively from files at the Medical University of Vienna, Department of Obstetrics and Gynecology, the Landeskrankenhaus Klagenfurt, Department of Obstetrics and Gynecology, and the Innsbruck Medical University, Department of Obstetrics and Gynecology. Six hundred five consecutive patients (Medical University of Vienna: n = 388, Landeskrankenhaus Klagenfurt: n = 79, Innsbruck Medical University: n = 138) with histological confirmed endometrial cancer undergoing surgery between December 1995 and January 2005 were included in our study. Five hundred forty three patients with endometrioid endometrial cancer were enrolled in all further analysis. Other histological subtypes were excluded from further analysis.

Based on patients' history and on the clinical examination performed by the referring physician and at time of referral to hospital, patients were classified into two groups according to presence (symptomatic patients) or absence (asymptomatic patients) of postmenopausal bleeding. Asymptomatic patients were defined as neglecting a type of bleeding in the previous 12 months, as having no signs of bleeding seen during the gynecological examination performed by the referring physician and at time of referral to hospital, and as not having any other symptoms indicative for endometrial cancer. Patients' personal history has been performed in the participating study centers in a standardized way. One of the most important questions was presence/absence of postmenopausal bleeding. This has been clearly documented in all patients' files.

### Clinical management

A guideline by the Austrian Society of Gynecology and Obstetrics (OEGGG) and the Austrian Society of Gynecologic Oncology (AGO) recommends endometrial sampling in cases of a postmenopausal bleeding or an endometrial thickness > 11 mm in asymptomatic women [10]. Asymptomatic women included in our study are real asymptomatic cases detected by "quasi-screening" and did not seek care for other reasons. Diagnosis of endometrial cancer was established by hysteroscopy, dilatation and curettage. Subsequently, patients were treated by hysterectomy, bilateral salping-oophorectomy, pelvic and in selected cases paraaortic lymphadenectomy. In cases of lymph node metastases, postoperative radiotherapy was applied according to standardized treatment protocols. Histological staging and grading was performed according to the current International Federation of Gynecology and Obstetrics (FIGO) classification on basis of the final pathologic evaluation of hysterectomy specimen.

Patients were followed up in regular intervals after surgery, including inspection, vagino-rectal and screening for serum tumor marker evaluation. In cases of clinically suspicious findings and/or tumor marker elevation computed tomography was performed. The same protocols for treatment and follow-up were used in all participating centers.

### Statistical analysis

Values are given as means (standard deviation [SD]) for evenly distributed values. Metric parameters were compared using Student's t-test. All data a Student's t-test was used for, were normally distributed. Nominal parameters were compared using Chi square test. Survival probabilities were calculated by a univariable Cox regression analysis (metric variables) or a product limit method of Kaplan and Meier using the log-rank test (categorical variables). A multivariable Cox regression model was performed comprising tumor stage (FIGO I vs. FIGO II vs. FIGO III vs. FIGO IV), tumor grade (G1 vs. G2 vs. G3), age at diagnosis, and presence/absence of irregular bleeding. The results were analyzed for the endpoint of disease-free and overall survival. Survival times of patients disease-free or still alive were censored with the last follow-up date. The proportional hazards assumption has been checked and found not to be violated. P-values of < 0.05 were considered statistically significant. We used the statistical software SPSS 11.0 for Windows (SPSS 11.0, SPSS Inc., Chicago, IL) for statistical analysis.

### Institutional review board

The present study was approved by our institutional review board, the Ethics-Committee of the Medical University of Vienna and the Vienna General Hospital - AKH.

## Results

**Figure 1:**
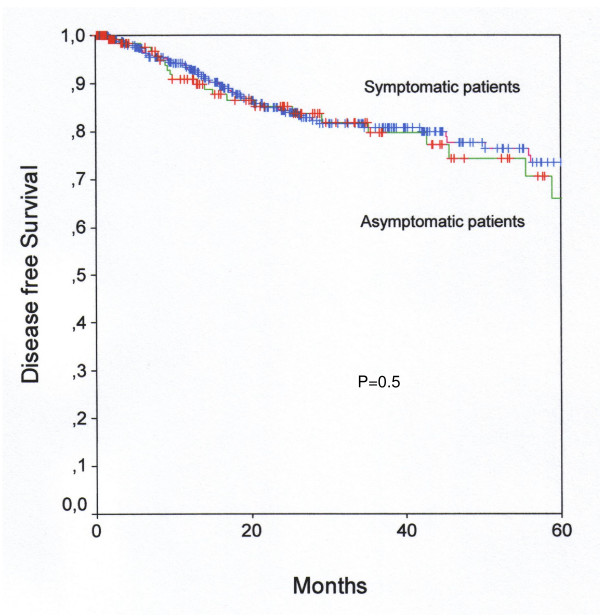
**Disease free survival**. Kaplan-Meier curve for disease free survival of asymptomatic and symptomatic patients with endometrioid endometrial cancer.

**Figure 2:**
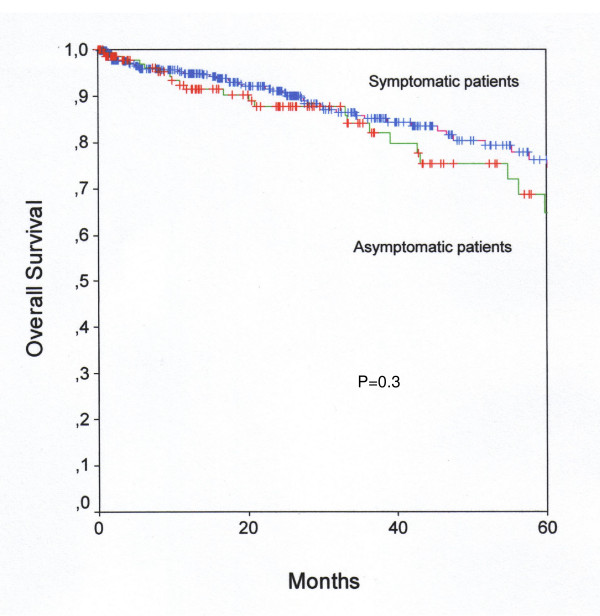
**Overall Survival**. Kaplan-Meier curve for overall survival of asymptomatic and symptomatic patients with endometrioid endometrial cancer.

Patients' characteristics of asymptomatic/symptomatic patients with endometrial cancer are shown in Table 1. Asymptomatic patients with endometrial cancer had a significantly higher rate of well differentiated tumors. FIGO tumor stages and patients' age were evenly distributed. In univariable and multivariable survival analysis, tumor stage, tumor grade, and patients' age at diagnosis were associated with disease free and overall survival (Table 2, Table 3). In a multivariable survival analysis presence/absence of a postmenopausal bleeding prior to diagnosis was not associated with disease free or overall survival (Table 3). Kaplan-Meier curves for the influence of presence/absence of a postmenopausal bleeding on disease-free (Figure 1) and overall (Figure 2) survival are shown.

**Table 1:** Patients' characteristics.

Parameter	Asymptomatic patients (No.[%])	Symptomatic patients (No. [%])	P
**Number of patients**	149	456	
**Patients with endometrioid endometrial cancer**	133	410	
**Age at first diagnosis (years SD )**	66.3 (10.3)	67.1 (11.2)	0.9*
**Tumor stage**			0.07^†^
I	100 (75.2)	286 (69.8)	
II	9 (6.8)	40 (9.8)	
III	15 (11.2)	71 (17.2)	
IV	9 (6.8)	13 (3.2)	
**Tumor grade**			0.006^†^
G1	74 (55.7)	170 (41.5)	
G2	45 (33.8)	153 (37.3)	
G3	14 (10.5)	87 (21.2)	
**Patients with follow-up information available**	133	409	
**Time of follow-up (months [SD])**	23.7 (24.03)	28.5 (25.76)	0.06*
**Recurrence status**			
Patients with recurrent disease	14 (10.5)	55 (13.4)	0.2^†^
Time to recurrent disease (months [SD])	18.5 (16.9)	20.4 (17.6)	0.7*
**Status at last observation**			0.7^†^
Alive with no evidence of disease	115 (86.5)	341 (83.4)	
Stable disease	0	3 (0.7)	
Progressive disease	8 (6.0)	34 (8.3)	
Dead as a result of disease	9 (6.8)	25 (6.1)	
Dead as a result of other causes	1 (0.7)	6 (1.5)	

SD = standard deviation, * two sided student's t-test, ^† ^Chi squared test

**Table 2:** Univariable survival analysis in patients with endometrial cancer.

	Disease free survival		Overall survival	
		
	HR (95% CI)	P	HR (95% CI)	P
Tumor stage (FIGO I vs. II vs. III vs. IV)	-	< 0.001*	-	< 0.001*
Tumor grade (G1 vs. G2 vs. G3)	-	< 0.001*	-	< 0.001*
Age at diagnosis	1.03 (1.01-1.05)	0.003^†^	1.05 (1.03-1.08)	< 0.001^†^

* Kaplan-Meier analysis, ^† ^univariable Cox-Regression model, HR (95% CI) = Hazard Ratio (95% Confidence Interval)

**Table 3:** Multivariable survival analysis in patients with endometrial cancer.

	Disease free survival*		Overall survival*	
		
	HR (95% CI)	P	HR (95% CI)	P
Tumor stage (FIGO I vs. II vs. III vs. IV)	1.9 (1.5-2.5)	< 0.001	2.2 (1.7-2.8)	< 0.001
Tumor grade (G1 vs. G2 vs. G3)	1.8 (1.3-2.6)	0.001	1.6 (1.1-2.4)	0.008
Age at diagnosis	1.03 (1.01-1.06)	0.01	1.05 (1.03-1.08)	< 0.001
Bleeding vs. non bleeding	0.9 (0.5-1.6)	0.7	0.8 (0.5-1.4)	0.4

* multivariable Cox-Regression model, HR (95% CI) = Hazard Ratio (95% Confidence Interval)

## Discussion

We present data on the prognostic value of presence/absence of a postmenopausal bleeding prior to diagnosis in large series of patients with endometrial cancer in a multi-center study. In parallel to previous studies [9], we did not ascertain a significant difference in prognosis between asymptomatic and symptomatic patients.

Screening for gynecological malignancies has been advocated in order to diagnose diseases at earlier stage leading to earlier treatment with less side-effects and better prognosis [11]. To date, screening tools for endometrial cancer have not been identified. Transvaginal ultrasound has been suggested to detect endometrial cancer in an earlier stage before the onset of clinically recognizable symptoms [6]. Our study falls short of showing any statistically significant difference in disease-free and overall survival of affected patients.

Based on our study design we cannot extrapolate our results to a real screening situation. Although having no formally organized screening program in Austria, the majority of postmenopausal women routinely see their gynecologist once a year, i.e. opportunistic screening. Besides the PAP smear, a transvaginal sonography is routinely performed during this examination. Based on the currently available evidence [5], a guideline by the Austrian Society of Gynecology and Obstetrics (OEGGG) and the Austrian Society of Gynecologic Oncology (AGO) recommends endometrial sampling in cases of asymptomatic women and an endometrial thickness > 11 mm [10]. This might be the most likely reason for having only 3/4 of women with endometrial cancer presenting with postmenopausal bleeding.

Of note, our study was not set out to evaluate the advantages or disadvantages of a transvaginal ultrasound in the screening of endometrial cancer. Studies demonstrating a positive effect of performing routine endometrial cancer screening on overall mortality would possibly have to include hundreds of thousands of women based on the relatively low incidence of the disease, the high rate of symptomatic women in early stage disease, and the good prognosis of affected women. Due to high costs and missing data on the reliability of TVS as screening tool for endometrial cancer the realization of a study like this would be hard to justify [12,13]. We speculate that such large-scale studies will not be performed and completed in the near future.

Our study supports previously published studies indicating that tumor biology in asymptomatic patients was different than in symptomatic patients [8]. This possibly reflects a higher propensity of low-differentiated tumors to cause postmenopausal bleeding than well/moderate differentiated endometrial cancers.

Our study has strengths and weaknesses, namely the retrospective study design, the extraction of information from patients' files, the relatively low number of events due to a generally good prognosis of affected patients, and the relatively short time of follow up. Another interesting point to mention is the relatively high percentage of women having advanced FIGO stages, i.e., FIGO II-IV, and still not having any symptoms. This can be easily explained in patients with FIGO IIIA, but not for other stages. Of note, we double-checked all FIGO stages and found our numbers to be correct. This has to be considered when interpreting the results of our study.

## Conclusion

To conclude, asymptomatic patients with endometrial cancer undergoing surgery have a higher rate of well and moderate differentiated tumors compared to patients with a postmenopausal bleeding prior to diagnosis. The prognosis of both groups of patients was similar.

## Competing interests

The authors declare that they have no competing interests.

## Authors' contributions

V.S. initiated the design of the study with L.H. and A.R. and was involved in developing and conducting the study, wrote drafts of the paper and reviewed the manuscript. M.S., K.H-F., H.L. and N.C. obtained the data from medical records, prepared the data for analysis and participated in reviewing of the manuscript. S.P. carried out statistical analysis and participated in reviewing the manuscript. A.R. and L.H. initiated the study design with V.S., coordinated analysis, interpreted results and participated in the writing and reviewing of the paper. All authors have read and approved the final manuscript.

## Pre-publication history

The pre-publication history for this paper can be accessed here:

http://www.biomedcentral.com/1471-2407/9/460/prepub
